# The Synthetic Microbiology Caucus: a fresh channel for exploring new ideas, challenging conventional wisdom and fostering community projects

**DOI:** 10.1111/1751-7915.13331

**Published:** 2018-10-25

**Authors:** Víctor de Lorenzo, Philippe Marliére

**Affiliations:** ^1^ Systems Biology Program Centro Nacional de Biotecnología (CNB‐CSIC) Campus de Cantoblanco Madrid 28049 Spain; ^2^ ISSB Génopole Equipe Xénome 91030 Evry Cedex France

Contemporary synthetic biology was established as a field of scientific and technological research in the early 2000s by electrical engineers and computational scientists – not by biologists. This foundational cause had numerous effects that have propagated to this day. The first is the focus on the relational logic of biological objects as a way to both understand why they work as they do and to reshape them for a different role – using as a mindframe the traditional hierarchy of parts/devices/systems. The second is the involvement of a diverse and playful community of SynBio practitioners that unleashes its imagination and creativity for overcoming academic constraints and IP barriers, culminating in the iGEM competition^1^ and the do‐it‐yourself (DIY) subversion^2^. The third is the emphasis on applications: most SynBio‐related news that makes headlines are about the surprising possibilities brought about by the field, from the cheap production of artemisinin in yeast^3^ for fighting the scourge of malaria to the resurrection of Neanderthals^4^ together with their woolly mammoth^5^ pets. These angles bring novel perspectives and challenges to the more conventional fields of research in the life sciences and the biotechnological industries.

However, not everyone reacts positively to these developments. It is often the case that well‐established researchers counter SynBio with skepticism as a mere attempt to rename existing areas with claims of novelty and adoption of fashionable techno‐jargon that evokes more a dilettante's agenda than a rigorously designed plan for crafting unprecedented biological objects and assemblies. Furthermore, many biology researchers argue that rationally designing living systems with an acceptable degree of predictability is simply impossible. This is because of the inherent context‐dependency of biological devices, the unavoidable emergence of mutations and the ensuing lack of durability – at odds with the expected robustness of typical engineered objects. The SynBio aficionados prefer not to talk about problems and focus instead on innumerable opportunities. The SynBio skeptics enjoy putting the finger in the problems and postpone the prospects until fundamental issues have been completely solved.

That the two communities, that is SynBio enthusiasts and SynBio agnostics, hardly meet and engage in a constructive conversation has become, in our view, a major bottleneck for deploying both the applied and the fundamental agendas of the field. Thus, there is a clear and present need of channelling and publicising in the same forum the diverse views held by various communities that claim territory in the SynBio field, but in a format in‐between the traditional academic publication and the free‐flowing internet‐based discussion fora. To fulfil this need, Microbial Biotechnology is launching a new section called *The Synthetic Microbiology Caucus*, with the aim not only to bridge the gaps between the different views of SynBio but also to harness (the difference‐of‐potential between) them for generating new ideas and launch community projects.

Typical contributions will consist of short pieces (1000–1500 words, at most one display item) reporting not results, but new concepts, raising questions (including those challenging conventional wisdom or established tenets) and outlines for new applied and basic endeavours. The section will be run by two dedicated Editors – Pablo Iván Nikel (The Novo Nordisk Foundation Center for Biosustainability, Denmark^6^) and Wei Huang (Oxford University, UK^7^). They will decide on the suitability of the pieces either on the spot or pursuing the assistance of external reviewers – who will make their best to have a decision made within days. The section will thus be highly dynamic and pro‐ and con‐ views on the same matters will be welcome. Otherwise, contributions will be treated as regular submissions, to be uploaded through the Microbial Biotechnology webpage and, if accepted, will be citable as ordinary publications. Editors will ensure that the inputs match high scientific and technical standards and that all kinds of dialogue between contrasting views remain in the constructive – not narcissistically destructive – realm. Some pieces may be the prelude of full‐fledged manuscripts submitted to the journal through the customary channel.

What topics could be the subject of contributions to the Synthetic Microbiology Caucus? Quite a few! Just to mention some: metabolic schemes, genome recoding, evolutionary scenarios, biodesign software, synthetic ecosystems and planetary remediation, biological computation, microbial chassis benchmarking, whole‐cell *vs*. cell‐free systems, synthetic cells, critical views of published articles, nomenclature and standards, metaphors and jargon for clarifying concepts and an unbounded *et cetera*. Especially welcome will be the pieces that pinpoint fundamental (but often swept under the carpet) scientific bottlenecks that need to be addressed for unleashing the whole potential of Synthetic Biology in biotech applications. It is remarkable that one can find many such topics (and interesting arguments about them) spread out in a plethora of more or less informal discussion forums in social media (Twitter, Facebook) and in a large number of personal blogs, whereas they rarely make it to academic publications and thereby fail to gain the visibility that they often deserve. We hope that the new Microbial Biotechnology section will ensure that good ideas, even at a preliminary stage, reach out the right recipients and chances of practical implementation increase.

What do we want to achieve with the Synthetic Microbiology Caucus? By letting contributors communicate their thoughts in a flexible format, we expect to identify key but otherwise neglected research questions that may be picked up – by the same authors or by others – as the basis of new projects and experiments. Also, we would like this section to host a candid but productive conversation between SynBio enthusiasts and agnostics, along the lines mentioned before, to come to a realistic conclusion on how far we can get with biological engineering and what is to be done to make it a reality. Propositions to formalize the important theoretical and experimental endeavours of SynBio will be welcomed as well. Finally, by exposing the ideas of contributors coming from different locations of the SynBio landscape we envisage the emergence of working groups of interest that could result in large and very large collaborative projects.

The ultimate organisational goal that we pursue is to make the Caucus hosted in this journal the editorial instrument perpetuating and amplifying the momentum that SynBio has already brilliantly gained and make sure its creations reach out beyond the enchanted world of the laboratory.

All together, let's give life another chance.

## **Conflict of interest**

None declared.

## Notes

1 http://igem.org/

2 https://onlinelibrary.wiley.com/doi/abs/10.1111/1751‐7915.12715

3 https://www.nature.com/articles/nature12051

4 http://www.spiegel.de/international/zeitgeist/george‐church‐explains‐how‐dna‐will‐be‐construction‐material‐of‐the‐future‐a‐877634.html

5 https://www.newscientist.com/article/2121503‐can‐we‐grow‐woolly‐mammoths‐in‐the‐lab‐george‐church‐hopes‐so/

6
http://www.biosustain.dtu.dk/research/research-groups/systems-environmental-microbiology

7 http://www.eng.ox.ac.uk/synbiosc/people/professor‐wei‐huang

